# *CYP1A2* Genetic Variation, Coffee Intake, and Kidney Dysfunction

**DOI:** 10.1001/jamanetworkopen.2022.47868

**Published:** 2023-01-26

**Authors:** Sara Mahdavi, Paolo Palatini, Ahmed El-Sohemy

**Affiliations:** 1 Department of Nutritional Sciences, Temerty Faculty of Medicine, University of Toronto, Toronto, Ontario, Canada; 2 Department of Medicine, University of Padova, Padova, Italy

## Abstract

**Importance:**

Caffeine is detoxified by cytochrome P450 1A2 (CYP1A2), and genetic variation in *CYP1A2* impacts the rate of caffeine clearance. Factors that may modify the association between coffee intake and kidney disease remain unclear.

**Objective:**

To assess whether *CYP1A2* genotype modifies the association between coffee intake and kidney dysfunction.

**Design, Setting, and Participants:**

The Hypertension and Ambulatory Recording Venetia Study (HARVEST) was a prospective cohort study of individuals with stage 1 hypertension in Italy; HARVEST began on April 1, 1990, and follow-up is ongoing. The current study used data from April 1, 1990, to June 30, 2006, with follow-up of approximately 10 years. Blood pressure and biochemical data were collected monthly during the first 3 months, then every 6 months thereafter. Data were analyzed from January 2019 to March 2019. Participants were screened and recruited from general practice clinics. The present study included 1180 untreated participants aged 18 to 45 years with stage 1 hypertension; those with nephropathy, diabetes, urinary tract infection, and cardiovascular disease were excluded.

**Exposures:**

Coffee intake and *CYP1A2* genotype rs762551 were exposures analyzed over a median follow-up of 7.5 (IQR, 3.1-10.9) years.

**Main Outcomes and Measures:**

Albuminuria (defined as an albumin level of ≥30 mg/24 h) and hyperfiltration (defined as an estimated glomerular filtration rate of ≥150 mL/min/1.73 m^2^) were the primary outcomes as indicators of kidney dysfunction.

**Results:**

Among 1180 participants, genotyping, lifestyle questionnaires, and urine analysis data were obtained from 604 individuals (438 [72.5%] male) with a mean (SD) age of 33.3 (8.5) years and a mean (SD) body mass index (calculated as weight in kilograms divided by height in meters squared) of 25.4 (3.4). A total of 158 participants (26.2%) consumed less than 1 cup of coffee per day, 379 (62.7%) consumed 1 to 3 cups per day, and 67 (11.1%) consumed more than 3 cups per day. Genotype frequencies for rs762551 (260 participants [43.1%] with genotype AA, 247 participants [40.8%] with genotype AC, and 97 participants [16.1%] with genotype CC) did not differ between coffee intake categories. The level of risk of developing albuminuria, hyperfiltration, and hypertension, assessed by Cox regression and survival analyses, was not associated with coffee intake in the entire group or among fast metabolizers. The risks of albuminuria (adjusted hazard ratio [aHR], 2.74; 95% CI, 1.63-4.62; *P* < .001), hyperfiltration (aHR, 2.11; 95% CI, 1.17-3.80; *P* = .01), and hypertension (aHR, 2.81; 95% CI, 1.51-5.23; P = .001) increased significantly among slow metabolizers who consumed more than 3 cups per day.

**Conclusions and Relevance:**

In this study, the risks of albuminuria, hyperfiltration, and hypertension increased with heavy coffee intake only among those with the AC and CC genotypes of *CYP1A2* at rs762551 associated with slow caffeine metabolism, suggesting that caffeine may play a role in the development of kidney disease in susceptible individuals.

## Key Points

**Question:**

Does *CYP1A2* genotype modify the association between coffee intake and kidney dysfunction?

**Findings:**

In this cohort study involving a 7.5-year follow-up of 1180 untreated participants with stage 1 hypertension from the Hypertension and Ambulatory Recording Venetia Study, those with a genetic variant in *CYP1A2* who were slow metabolizers of caffeine were 2.7 times more likely to develop albuminuria, 2.5 times more likely to develop hyperfiltration, and 2.8 times more likely to develop hypertension with heavy coffee intake compared with low coffee intake. No associations were observed between coffee intake and albuminuria, hyperfiltration, or hypertension among fast metabolizers of caffeine.

**Meaning:**

These findings suggest that heavy coffee intake is associated with increases in the risk of kidney dysfunction among slow metabolizers of caffeine, who genetically comprise approximately half of the population, but not among fast metabolizers of caffeine.

## Teaser

This cohort study assesses whether *CYP1A2* genotype modifies the association between caffeine intake and kidney dysfunction among untreated individuals in Italy aged 18 to 45 years with stage 1 hypertension.

## Introduction

Preventable kidney disease is one of the leading causes of morbidity and mortality worldwide.^1^ Factors that may modify the association between coffee consumption and kidney disease remains unclear. Coffee is a major source of caffeine and the most widely consumed caffeinated beverage in the world. Some investigations have suggested that caffeine is associated with impaired kidney function in humans^2^ and adversely alters kidney tissue in animals and humans by stimulating glomerular remodeling and sclerosis,^3,4^ worsening hypertension^5,6^ and proteinuria,^7^ and accelerating preexisting chronic kidney failure.^8^ However, other studies have found that caffeine intake can slow the progression of diabetic nephropathy,^9^ while others have observed a lower risk of developing chronic kidney disease (CKD) with increasing coffee consumption.^10^ Some studies have reported an increased risk of cyst enlargement in patients with autosomal dominant polycystic kidney disease with increasing caffeine intake,^11^ while others have reported no consequences.^12,13^ Previous studies, however, have not accounted for the wide interindividual variation in metabolism of caffeine, which could modify any associations.

Caffeine is a central nervous system stimulant and the most commonly consumed psychoactive substance.^14^ Its mechanisms of action include inhibiting the phosphodiesterase enzyme, antagonizing adenosine receptors, and activating the ryanodine receptors with several actions on multiple organs.^15^ Caffeine has the potential to exert adverse effects on kidney function and structure by stimulating some of the key proliferative mechanisms involved in glomerular remodeling and sclerosis.^2,3,4,5,6,7,8^ In addition, because adenosine acts as a vasodilator to prevent hypoxic injury in the renal medulla,^16^ caffeine’s antagonistic action could induce hypoxic injury. However, data are inconclusive, with evidence ranging from coffee consumption being protective against chronic kidney disease^17,18,19,20^ to having no associations with^5,21,22^ or even accelerating kidney disease.^6,11,23^

More than 95% of caffeine is metabolized by cytochrome P450 1A2 (CYP1A2) and a common polymorphism in the *CYP1A2* gene has been reported to be significantly associated with caffeine metabolism.^24^ The rs762551 variant decreases enzyme activity and inducibility.^24,25^ Individuals with AC and CC genotypes are considered slow metabolizers, and those with the AA genotype are considered fast metabolizers.^26^ This polymorphism has been found to modify the association between coffee intake and the risk of myocardial infarction,^27^ hypertension,^28^ and impaired fasting glucose^29^ in a dose-dependent manner. In those studies,^27,28,29^ an increased risk was observed in slow metabolizers with increasing cups of coffee consumed per day, whereas in fast metabolizers, increasing cups of coffee per day was either associated with a lower risk, or no association was found. Those findings might explain some of the inconsistencies observed in reports of coffee intake and kidney disease; however, previous studies examining the association between coffee and the risk of kidney disease have not examined the role of *CYP1A2*. The objective of the present study was to assess whether *CYP1A2* genotype rs762551 modified the association between intake of caffeinated coffee and markers of kidney dysfunction.

## Methods

### Participants

This study was conducted with participants from the Hypertension and Ambulatory Recording Venetia Study (HARVEST), a prospective longitudinal study of untreated participants aged 18 to 45 years with stage 1 hypertension.^30^ HARVEST was conducted in 17 hypertension units in Italy beginning on April 1, 1990, and follow-up is ongoing. The current study used data from April 1, 1990, to June 30, 2006, with follow-up of approximately 10 years. Data were analyzed from January 2019 to March 2019. Detailed study methods have been reported elsewhere.^30^ The present study included 1180 male and female individuals aged 18 to 45 years who took part in HARVEST^30^; those with nephropathy, diabetes, urinary tract infection, and cardiovascular disease were excluded. A genetic substudy included 639 participants from 4 centers (Padova, Vittorio Veneto, San Daniele del Friuli, and Trento) who agreed to participate in the genetic study.^28^ Data on race and ethnicity were not empirically collected for this study, but the study population has been described as being of White-European ethnicity with the majority being of Italian origin. The ethics committee of the University of Padova approved the study, and the participants gave written informed consent to participate. Further details are provided in the eAppendix in Supplement 1. This study followed the Strengthening the Reporting of Observational Studies in Epidemiology (STROBE) guideline for cohort studies.

Details about the interview, lifestyle assessment, and criteria used for participant classification according to lifestyle have been reported elsewhere.^28,31,32^ Clinic blood pressure (BP) was the mean of 6 readings obtained during 2 visits to the clinic performed 2 weeks apart. Body mass index (BMI; calculated as weight in kilograms divided by height in meters squared) was considered as an index of adiposity.

### Follow-up Procedures

Blood samples were obtained on an annual basis for routine measures. Clinic BP and lifestyle habits were assessed monthly during the first 3 months of follow-up, then after 6 months and every 6 months thereafter. After baseline examination, participants were given general lifestyle information about nonpharmacological measures following the suggestions of the most current guidelines on the management of hypertension.^33,34,35,36^ To ensure homogeneous counseling by physicians participating in the study, training in current international guidelines was provided to them throughout the study duration. Coffee intake recommendations were not provided as a part of the intervention because coffee intake was not included in any of the available guidelines.

HARVEST participants were followed up until they developed sustained hypertension requiring antihypertensive treatment according to the guidelines available at the time.^33,34,35,36^ When patients developed sustained hypertension, the investigators performed a final clinical assessment including biochemical tests before they exited the study and pharmaceutical treatment was initiated. Participants who did not meet the criteria for treatment continued to be followed up at 6-month intervals. Routine blood tests, including assessment of creatinine levels, were performed at yearly intervals and at the end of the study. Of 1180 participants, 683 (57.9%) developed hypertension during a median follow-up of 6.1 (IQR, 2.5-9.9) years. This proportion was similar among participants included in the genetic substudy. Among participants who remained normotensive, the last available clinical assessment was used. All data used for the present study were collected from untreated patients.

The estimated glomerular filtration rate (eGFR) was calculated from creatinine clearance, which was computed from creatinine excretion in a 24-hour urine collection and a single measurement of serum creatinine, and the data were normalized by body surface area.^37^ The urinary albumin level was measured by a commercially available radioimmunoassay kit (H ALB kit–double antibody; Sclavo SpA). Results were expressed as milligrams per 24 hours and were transformed logarithmically. The albumin excretion rate (AER) was available for 954 participants in the parent study. The adequacy of the 24-hour urine collections was assessed by self-report of missed or spilled collections as well as by creatinine excretion per kilogram of body weight.^38^ Participants were defined as normofilterers or hyperfilterers according to whether their eGFR was lower than 150 mL/min/1.73 m^2^ or 150 mL/min/1.73 m^2^ or higher, respectively.^39^ Albuminuria was defined as an albumin level of 30 mg/24 h or higher, and hypertension was defined as a clinic BP of 140/90 mm Hg or higher.

### Coffee Consumption and Genotyping

Participants completed questionnaires about their medical history, family history of hypertension, physical activity, and dietary habits, including coffee intake, alcohol use, and cigarette smoking. Detailed methods for these procedures and their validity have been reported elsewhere.^28,29,31,32^ Briefly, coffee consumption was defined according to the number of caffeine-containing cups of coffee consumed per day. The caffeine content per cup was defined as 100 mg of Italian espresso coffee, which was the most abundantly consumed type of coffee by the HARVEST participants.^40^ Decaffeinated coffee, tea, and other caffeinated drinks were not taken into account in the present study because they were rarely consumed in these areas of Italy.^41^ Coffee consumption was then categorized into 3 groups: low coffee intake (<1 cup per day), moderate coffee intake (1-3 cups per day), and heavy coffee intake (>3 cups per day) based on classification used in previous analyses.^31,42,43^ DNA was extracted from whole blood and genotyped for the rs762551 polymorphism in *CYP1A2* as previously described.^28^

### Statistical Analysis

The present analysis included 604 of 1180 participants who had biochemical data and information on lifestyle habits and genotype available (both at baseline and final assessments) and at least 6 months of follow-up. Participants were grouped into slow (AC and CC genotypes) or fast (AA genotype) metabolizers according to *CYP1A2* genotype and habitual consumption of coffee (low, moderate, or heavy). The distribution of participant characteristics was compared by genotype and across groups of coffee consumption using analysis of variance for continuous variables and χ^2^ testing for categorical variables.

The cumulative incidence of albuminuria, hyperfiltration, and hypertension associated with coffee consumption at baseline was calculated using Kaplan-Meier analysis for each outcome separately. The difference in albuminuria, hyperfiltration, and hypertension incidences between coffee drinking categories was assessed using the log-rank test. Coffee intake was also modeled as a time-dependent categorical variable in a Cox proportional hazards analysis adjusted for possible confounding variables derived from significant associations of univariate analyses with the respective outcome measure. The variables found to be associated with outcome on the univariate survival analysis and/or considered to have potential prognostic importance were age, sex, BMI, baseline plasma glucose level, and serum triglyceride levels. Separate models were created to examine the associations with coffee consumption among slow and fast metabolizers. Estimates of relative risk (hazard ratios [HRs]) and corresponding 95% CIs for categories of *CYP1A2* genotype and coffee consumption were computed from Cox regression models that were adjusted for relevant variables in each model reported.

The threshold for statistical significance was 2-tailed *P* < .05. Analyses were performed using IBM SPSS Statistics software, version 27 (IBM Corporation).

## Results

### Population Characteristics

Among 604 participants (438 [72.5%] male) who completed genetic testing and lifestyle questionnaires and provided urine analysis data, the mean (SD) age was 33.3 (8.5) years, and the mean (SD) BMI was 25.4 (3.4). Genotype frequencies for rs762551 (260 participants [43.1%] with genotype AA, 247 participants [40.8%] with genotype AC, and 97 participants [16.1%] with genotype CC) did not differ between coffee intake categories. Among the entire cohort, 158 participants (26.2%) had low coffee intake (<1 cup per day), 379 (62.7%) had moderate coffee intake (1-3 cups per day), and 67 (11.1%) had heavy coffee intake (>3 cups per day). Compared with participants with low coffee intake, those with moderate and heavy intake were older (mean [SD], 33.9 [8.1] years for moderate intake and 37.2 [6.8] years for heavy intake vs 30.7 [9.3] years for low intake; *P* < .001) and had higher BMI (mean [SD], 25.6 [3.4] for moderate intake and 26.0 [4.0] for high intake vs 24.6 [3.3] for low intake; *P* = .003). The proportion of those with slow metabolism genotypes (AC and CC) did not differ significantly between the coffee intake categories (58.9% for low intake, 54.9% for moderate intake, and 64.5% for heavy intake; *P* = .31).

Overall, 4 (6.3%) of 64 participants with hyperfiltration also had albuminuria, and 5 (16.7%) of 30 participants with albuminuria had hyperfiltration. At baseline, clinic systolic BP was lower in fast metabolizers compared with slow metabolizers (mean [SD], 144.64 [10.15] mm Hg vs 146.65 [10.62] mm Hg; *P* = .02). Fasting glucose at baseline was also marginally lower in fast metabolizers vs slow metabolizers (mean [SD], 91.93 [10.09] mg/dL vs 93.99 [11.60] mg/dL; *P* = .03). No other participant characteristics were different at baseline and follow-up according to genotype (Table^44^).

**Table.** Characteristics of Study Participants by *CYP1A2* Genotype

Characteristic	*CYP1A2* genotype, No. (%)		*P* value
	AA (n = 260)	AC and CC (n = 344)	
Age, mean (SD), y	32.9 (8.5)	33.7 (8.5)	.23
Sex			
Female	70 (26.9)	96 (27.9)	.77
Male	190 (73.1)	248 (72.1)	
Coffee intake, cups/d			
<1	62 (23.8)	96 (27.9)	.56
1-3	172 (66.2)	207 (60.2)	.18
>3	26 (10.0)	41 (11.9)	.20
Drinks alcohol	107 (41.2)	134 (39.0)	.47
Smokes	55 (21.2)	62 (18.0)	.34
Follow-up period, mean (SD), d	2819 (1702)	2631 (1600)	.16
BMI, mean (SD)			
Baseline	25.3 (3.4)	25.4 (3.5)	.77
Follow-up	26.1 (3.9)	25.8 (3.4)	.30
eGFR, mean (SD), mL/min/1.73 m^2^^a^			
Baseline	101.42 (17.06)	101.89 (17.59)	.74
Follow-up	120.25 (29.40)	121.50 (29.38)	.69
AER, mean (SD), mg/24 h			
Baseline	12.96 (30.23)	11.10 (22.14)	.79
Follow-up	19.03 (56.62)	13.80 (25.16)	.57
Systolic BP, mean (SD), mm Hg			
Baseline clinical	144.64 (10.15)	146.65 (10.62)	.02
Follow-up	142.76 (12.95)	145.66 (14.32)	.009
Diastolic BP, mean (SD), mm Hg			
Baseline clinical	93.28 (5.59)	93.60 (5.39)	.48
Follow-up	93.52 (9.59)	94.25 (10.87)	.39
Glucose, mean (SD), mg/dL			
Baseline	91.93 (10.09)	93.99 (11.60)	.03
Follow-up	94.80 (14.20)	94.50 (12.93)	.81
HDL cholesterol, mean (SD), mg/dL			
Baseline	51.97 (14.22)	52.07 (14.44)	.94
Follow-up	55.73 (16.31)	53.52 (13.99)	.11
Triglycerides, mean (SD), mg/dL			
Baseline	113.73 (81.35)	114.79 (71.44)	.40
Follow-up	119.37 (90.12)	118.98 (75.26)	.88
Epinephrine, mean (SD), pg/mL			
Baseline	104.14 (18.18)	104.85 (18.13)	.71
Follow-up	101.92 (20.58)	103.29 (22.27)	.55
Heart rate, mean (SD), beats/min			
Baseline	74.55 (10.18)	74.97 (9.73)	.60
Follow-up	72.05 (9.74)	71.47 (9.19)	.46

Abbreviations: AER, albumin excretion rate; BMI, body mass index (calculated as weight in kilograms divided by height in meters squared); BP, blood pressure; eGFR, estimated glomerular filtration rate; HDL, high-density lipoprotein.

SI conversion factors: To convert glucose to mmol/L, multiply by 0.0555; to convert HDL cholesterol to mmol/L, multiply by 0.0259; to convert triglycerides to mmol/L, multiply by 0.0113; to convert epinephrine to pmol/L, multiply by 5.459.

^a^ Baseline eGFR was calculated using the 2009 Chronic Kidney Disease Epidemiology Collaboration creatinine equation.^44^

### Follow-up

Participants were followed up for a median of 7.5 (IQR, 3.1-10.9) years. During the follow-up, coffee intake habits remained the same for 581 participants. Among those who reported changing their coffee intake habits, 11 increased consumption and 12 decreased consumption. Recategorizing these participants did not materially alter any of the results; therefore, all participants remained in their baseline categories. Prevalence of albuminuria did not differ according to genotype at baseline (16 participants [6.2%] who were fast metabolizers and 21 participants [6.1%] who were slow metabolizers). However, at follow-up, this proportion increased to 8.1% (28 participants) of slow metabolizers, while it remained unchanged in the fast metabolizers. Hyperfiltration was not prevalent at baseline; however, at follow-up, 16.9% of fast metabolizers (44 participants) and 18.0% of slow metabolizers (62 participants) had an eGFR higher than 150 mL/min/1.73 m^2^. A higher prevalence of hypertension was observed in slow metabolizers vs fast metabolizers at baseline (204 participants [59.7%] vs 122 participants [46.9%]; *P* = .002), but these differences were no longer statistically significant at follow-up (175 participants [50.9%] vs 117 participants [45.0%]; *P* = .15).

### Association of Genotype and Coffee Intake With Albuminuria

**Figure 1.**
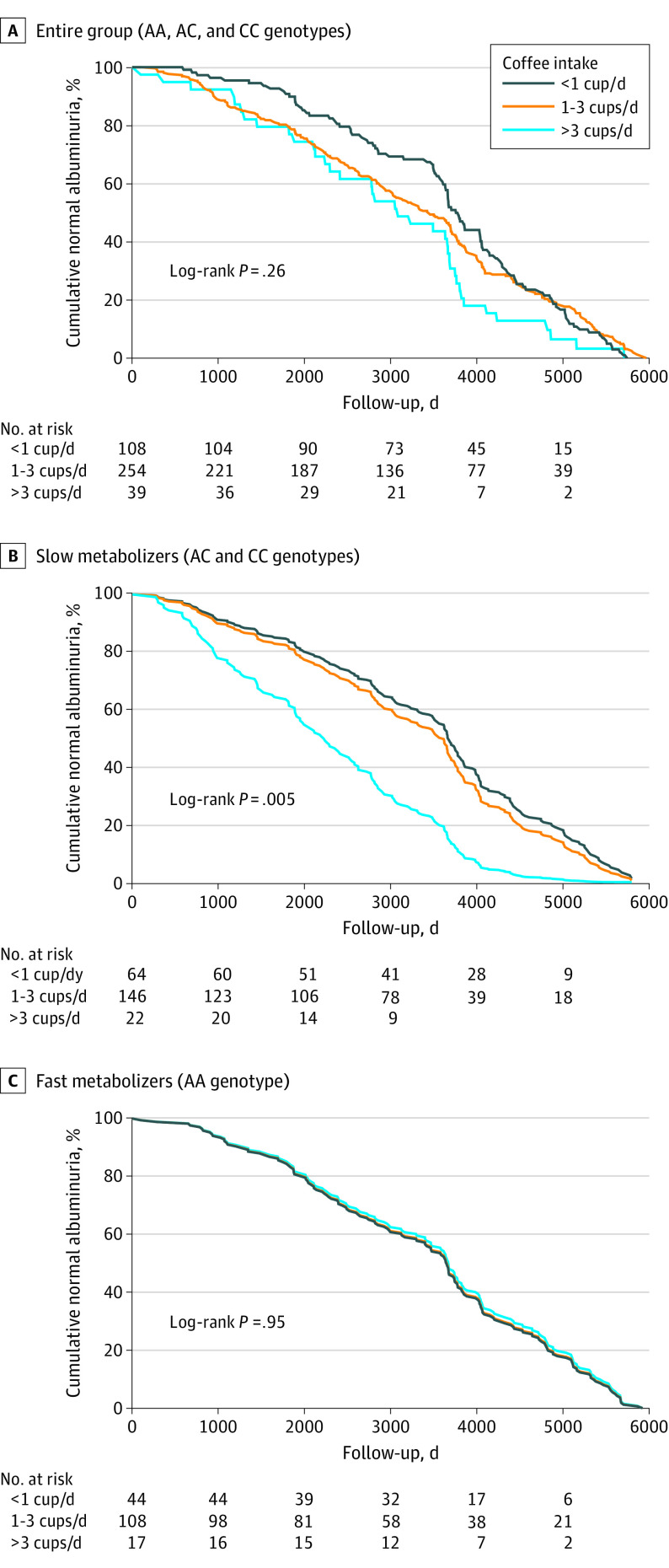
Kaplan-Meier Survival Curves of the Risk of Albuminuria by Coffee Intake and *CYP1A2* Genotype Among 407 participants. Normal albuminuria was defined as albumin level lower than 30 mg/24 h. Median follow-up was 7.5 (IQR, 3.1-10.9) years.

A total of 407 participants had AER records both at baseline and end of follow-up. Albuminuria (AER >30 mg/24 h) was detected in 28 participants (6.9%) by study end, and 52 participants (12.8%) had elevated AER (16-29 mg/24 h). The risk of developing albuminuria was not associated with coffee intake in the entire group (Figure 1A). However, when analyses were stratified by genotype, the risk of developing albuminuria in slow metabolizers increased significantly for heavy coffee drinkers (Cox regression model: unadjusted HR, 2.72 [95% CI, 1.64-4.50; *P* < .001]; model adjusted for age, sex, baseline BMI, baseline clinic systolic BP, baseline eGFR: HR, 2.74 [95% CI, 1.63-4.62; *P* < .001) (Figure 1B). In fast metabolizers, no association between coffee intake and albuminuria was observed (Figure 1C). Kaplan-Meier analysis of the slow metabolizers found that heavy coffee intake was associated with albuminuria 2.8 years earlier than low coffee intake.

### Association of Genotype and Coffee Intake With Hyperfiltration

**Figure 2.**
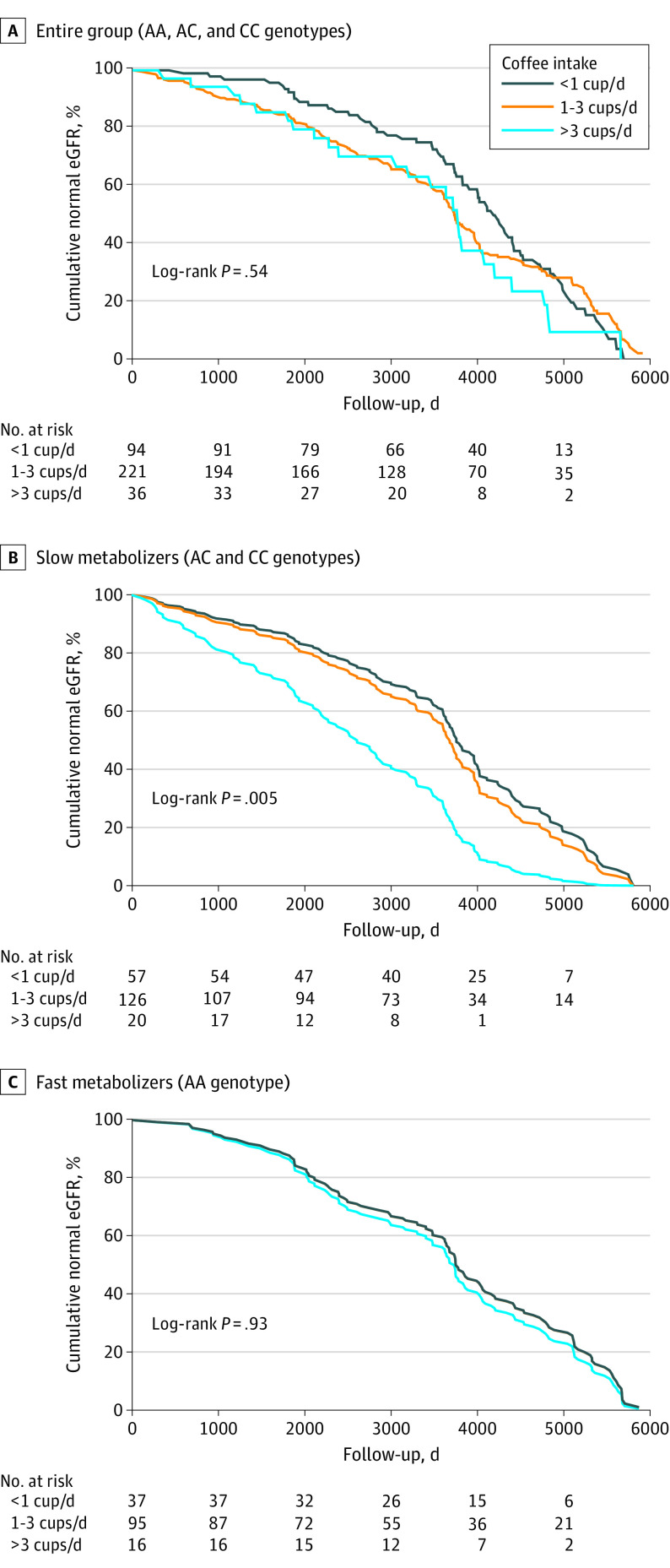
Kaplan-Meier Survival Curves of the Risk of Hyperfiltration by Coffee Intake and *CYP1A2* Genotype Among 351 participants. Normal estimated glomerular filtration rate (eGFR) was defined as 90 to 150 mL/min/1.73 m^2^. Median follow-up was 7.5 (IQR, 3.1-10.9) years.

A total of 351 participants had eGFR records both at baseline and end of follow-up. Hyperfiltration (eGFR >150 mL/min/1.73 m^2^) was detected in 61 participants (17.4%) by study end, and 73 participants (20.8%) had an elevated eGFR (125-150 mL/min/1.73 m^2^). The risk of developing hyperfiltration was not associated with coffee intake in the entire group (Figure 2A). When stratified by genotype, the risk of developing hyperfiltration in slow metabolizers increased significantly with heavy coffee drinking (Cox regression model: unadjusted HR, 2.47 [95% CI, 1.41-4.34; *P* = .002]; model adjusted for age, sex, baseline BMI, baseline clinic systolic BP, and baseline urinary albumin: HR, 2.11 [95% CI, 1.17-3.80; *P* = .01]) (Figure 2B). In fast metabolizers, no association between coffee intake and hyperfiltration was observed (Figure 2C). Kaplan-Meier analysis of the slow metabolizers found that heavy coffee intake was associated with hyperfiltration 4.9 years earlier than low coffee intake.

### Association of Genotype and Coffee Intake With Hypertension

**Figure 3.**
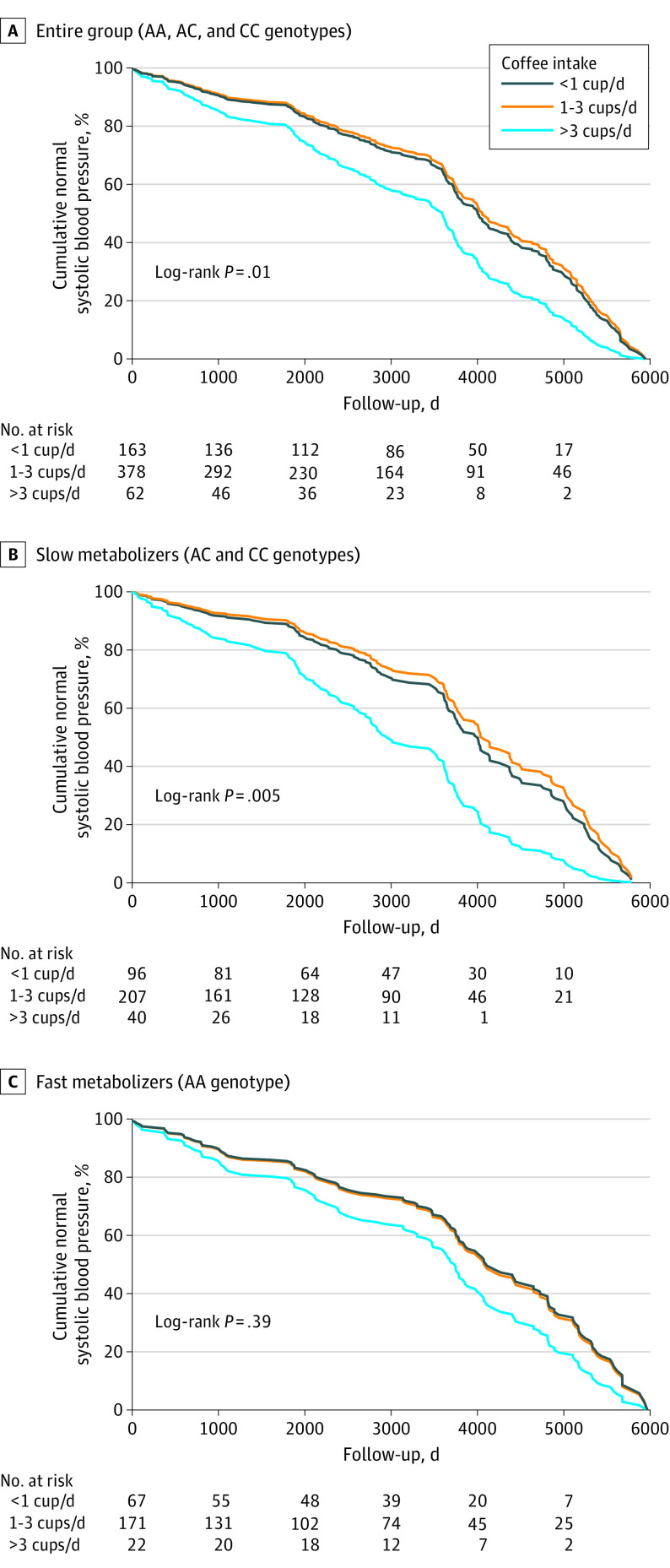
Kaplan-Meier Survival Curves of the Risk of Hypertension by Coffee Intake and *CYP1A2* Genotype Among 604 participants. Normal systolic blood pressure was defined as lower than 140 mm Hg. Median follow-up was 7.5 (IQR, 3.1-10.9) years.

A total of 604 participants had clinic systolic and diastolic BP records both at baseline and end of follow-up. Hypertension (clinic BP >140/90 mm Hg) was detected in 292 participants (48.3%) by study end. Heavy coffee drinkers had a 60% higher risk of developing hypertension compared with nondrinkers (HR, 1.60; 95% CI, 1.08-2.37; *P* = .02). The risk of developing hypertension was associated with coffee intake in the entire group (Figure 3A). However, when stratified by genotype, the risk of developing hypertension in slow metabolizers increased significantly with heavy coffee drinking (Cox regression model: unadjusted HR, 2.39 [95% CI, 1.34-4.29; *P* = .003]; model adjusted for age, sex, baseline BMI, baseline eGFR, and urinary albumin: HR, 2.81 [95% CI, 1.51-5.23; *P* = .001]) (Figure 3B), while no effect of coffee intake on hypertension was observed in fast metabolizers (Figure 3C). Kaplan-Meier analysis of the slow metabolizers found that heavy coffee intake was associated with hypertension 1.5 years earlier than low coffee intake.

## Discussion

The findings of this cohort study revealed that consuming more than 3 cups of coffee per day was associated with increases in albuminuria, hyperfiltration, and hypertension in slow metabolizers but not fast metabolizers. A previous study^38^ reported no association between coffee consumption and albuminuria in the HARVEST cohort. However, once the results were analyzed according to genotype in the present study, the risk of developing albuminuria was almost 3-fold higher in heavy coffee drinkers who were slow metabolizers compared with those consuming less than 1 cup per day, while no associations were observed among fast metabolizers. According to 2 meta-analyses,^20,45^ coffee and caffeine may have beneficial implications for kidney outcomes^20^ and may be associated with decreases in all-cause mortality in those with CKD.^45^ However, there were several limitations in the studies included in those meta-analyses, and none of them considered individual genetic differences in caffeine metabolism.

In the present study, coffee was defined as Italian espresso, which is a homogeneous form of caffeinated beverage that corresponds to the equivalent of approximately 100 mg of caffeine per cup. In a recent meta-analysis^20^ that reported a protective benefit of coffee intake for kidney outcomes, coffee consumption was reported as infrequently as less than 1 cup per week to an upper limit of more than 2 cups per day. Another meta-analysis,^10^ which found no association between coffee intake and CKD outcomes, used binary coffee intake categories comprising those who consumed more than 1 cup of coffee per day or those who consumed less than 1 cup per day. In the present study, we observed a significant coffee-gene interaction with kidney outcomes only when comparing those consuming more than 3 cups per day with those consuming less than 1 cup per day.

In most studies, increased caffeine intake has been associated with eGFR and thus with improvements in CKD,^20,21^ although these associations were often inconsistent. Another important consideration missed in prior studies is acute vs long-term exposure to caffeine. Acute exposure to caffeine will likely increase glomerular filtration temporarily; however, long-term exposure might have different outcomes. Hence, the association between coffee intake and kidney dysfunction may vary according to the etiology of kidney disease, stages of kidney disease, exposure time, and genetic differences. Increased eGFR higher than 150 mL/min/1.73 m^2^, also called hyperfiltration, is a proposed mechanism for kidney injury in several clinical conditions.^2^ Certain kidney diseases may also manifest as hyperfiltration in early stages, and hyperfiltration itself is often misclassified as an indication of optimal kidney function. However, those in the highest quartile of eGFR are at significantly higher risk of death, heart failure, cardiopulmonary events,^46^ and subsequent kidney disease that eventually manifests as declining eGFR.^39^ It has been suggested that caffeine has implications for kidney function through nonselective binding to adenosine receptors, which then modulate changes in eGFR via diuresis and natriuresis.^47^

Hyperfiltration has been identified as a factor associated with CKD in individuals with hypertension^46^ and diabetes^48^ as well as those with prediabetes and prehypertension.^48,49^ In patients diagnosed with type 2 diabetes, a higher eGFR was associated with rapid kidney function decline and subsequently impaired eGFR.^48^ Coffee consumption has been associated with kidney hyperfiltration in several studies,^2,50^ but others have reported an inconsistent association.^21,51^ Hyperfiltration is reversible and has been suggested as an early clinical measure of kidney dysfunction and a factor associated with albuminuria.^2,50^^,^ Albuminuria itself is associated with cardiovascular disease and kidney disease both in the general population^52,53^ and in populations with chronic illness.^48,53,54,55^ In a large study of patients with heart failure,^56^ elevated albumin excretion was an important marker of disease progression, independent of diabetes, hypertension, or serum creatinine. In the current study, albuminuria and hyperfiltration occurred independent of each other as outcome markers of kidney dysfunction in most of the study population. Only 4 of 64 participants with hyperfiltration (6.3%) also had albuminuria, and only 5 of 30 participants with albuminuria (16.7%) had hyperfiltration. Furthermore, when respective models were adjusted for urinary albumin and creatinine clearance, no material changes in the results were observed. Therefore, the association of hyperfiltration and albuminuria with lifestyle factors such as caffeine consumption may aid in risk-reduction strategies.

Since kidney disease often codevelops with other comorbidities, some studies in older adults might observe coffee being protective indirectly by being protective against other known comorbidities such as type 2 diabetes. In a large cohort of South Korean participants, coffee consumption was only protective against kidney disease in older diabetic women, and no association was found between coffee intake and kidney disease in other groups.^51^ In the present study of young adults with prehypertension to moderate hypertension who only received lifestyle intervention to manage hypertension, we observed an association between heavy coffee consumption and hyperfiltration that was modified by *CYP1A2* genotype. Previous studies have either found an inverse association between coffee consumption and CKD outcomes^19,20^ or an increased risk of kidney disease with coffee intake.^57^ However, no previous studies have accounted for genetic differences when assessing the association between coffee intake and CKD outcomes.

### Limitations

This study has several limitations. One limitation is that the parent HARVEST study was performed in 17 hypertension units in Italy, yet the present study was conducted in 4 units and did not account for clustering of participants across different units. Nevertheless, participants were from similar ethnic backgrounds, and no genetic or lifestyle differences were noted in the different groups. The observational nature of the current study could be viewed as a possible limitation, so future intervention studies that restrict caffeine intake among slow metabolizers would help provide more direct evidence to confirm these findings.

## Conclusions

In the present cohort study, caffeinated coffee intake was associated with increases in the risks of albuminuria, hyperfiltration, and hypertension only among slow metabolizers of caffeine, suggesting that caffeine may play a role in the development of kidney disease in susceptible individuals. These findings have implications for DNA-based interventions, such as precision nutrition recommendations, to reduce the risk of kidney disease.

## Notes

Supplement 1. **eAppendix.** Participants, Baseline Procedures, Kidney and Adrenal Function, and Data Analysis

Supplement 2.
Data Sharing Statement
