# Supplementary material for: CYP1A2 Genetic Variation, Coffee Intake, and Kidney Dysfunction
Source: JAMA Netw Open. 2023 Jan 26;6(1):e2247868. doi: 10.1001/jamanetworkopen.2022.47868 (PMC9880799; doi:10.1001/jamanetworkopen.2022.47868)
Supplement: Supplement 1. — eAppendix. Participants, Baseline Procedures, Kidney and Adrenal Function, and Data Analysis [file jamanetwopen-e2247868-s001.pdf]

# Supplemental Online Content

Mahdavi S, Palatini P, El-Sohemy A. *CYP1A2* genetic variation, coffee intake, and kidney dysfunction. *JAMA Netw Open*. 2023;6(1):e2247868.
doi:10.1001/jamanetworkopen.2022.47868

**eAppendix.** Participants, Baseline Procedures, Kidney and Adrenal Function, and Data Analysis

This supplemental material has been provided by the authors to give readers additional information about their work.

© 2023 Mahdavi S et al. *JAMA Network Open*.

**eAppendix.** Participants, Baseline Procedures, Kidney and Adrenal Function, and Data Analysis

*Participants*

Briefly, recruitment of patients was completed with the collaboration of the local general practitioners who were trained in screening methodology during local meetings. The study participants were never treated but screened for stage 1 hypertension and recruited from offices of general practitioners. All qualified patients willing to participate in the study were eligible for recruitment and were sent to the referral centers. Patient files, blood and urine samples were periodically collected by five monitors and taken to the coordinating office in Padova, where they were processed. Patients with nephropathy, (eGFR <60 ml/min/1.73 $m^2$), diabetes mellitus, urinary tract infection or cardiovascular disease (CVD) were excluded.

*Baseline Procedures*

Subject characteristics from the genetic sub-study were like those of the rest of the study population. In particular, the frequency of coffee drinkers (74%) and abstainers (26%) was similar in the two subgroups. Enrolment of subjects with the AA genotype versus those with AC or CC genotype as well as coffee drinkers versus abstainers was equally distributed throughout the years and centers. At baseline, all subjects underwent physical examination, anthropometry, blood chemistry, resting electrocardiogram, office and 24-hour blood pressure (BP) and 24-hour urine collection.

*Kidney and Adrenal Function*

Subjects were given verbal instructions on the collection of urine, which was performed during the 24-hour BP recordings. All collections were made in an unrestricted manner and no dietary restrictions were imposed. Immediately after completion, volumes were measured, and aliquots of urine (10 ml) were taken from the 24-hour collection and stored in glass tubes at −20 °C. Thereafter, urine specimens were sent to the coordinating office in Padova.

*Data Analysis*

The distribution of subject characteristics was compared across groups of coffee consumption and by genotype using ANOVA for continuous variables and chi-square test for categorical variables. The cumulative incidence of albuminuria, hyperfiltration, and hypertension associated with coffee consumption at baseline was calculated using Kaplan–Meier analysis for each outcome separately. The difference in albuminuria, hyperfiltration, hypertension incidences between coffee drinking categories was tested using the log-rank test. Coffee intake was also modeled as a time-dependent categorical variable in Cox proportional hazards analysis adjusting for possible confounding variables derived from significant associations of univariate analyses with the respective outcome measure. The variables found to be associated with outcome at univariate survival analysis and/or considered to be of prognostic importance were age, sex, BMI, baseline plasma glucose and serum triglycerides. Separate models were created to examine the associations with coffee consumption among slow and fast metabolizers. Estimates of relative risk (hazard ratio) and corresponding 95% confidence intervals (CIs) for categories of coffee consumption and CYP1A2 genotype were computed from the Cox regression models, adjusting for the same variables as above. Level of significance 0.05 was used in the inferential analysis, with p-values < 0.05 considered statistically significant. Analyses were performed using SPSS version 27 (SPSS Inc., Evanston, IL, USA).

© 2023 Mahdavi S et al. *JAMA Network Open*.
