# Supplementary material for: CYP1A2 Genetic Variation, Coffee Intake, and Kidney Dysfunction
Source: JAMA Netw Open. 2023 Jan 26;6(1):e2247868. doi: 10.1001/jamanetworkopen.2022.47868 (PMC9880799; doi:10.1001/jamanetworkopen.2022.47868)
Supplement: Supplement 2. — Data Sharing Statement [file jamanetwopen-e2247868-s002.pdf]

Data Sharing Statement

Data
Data available: Yes
Data types: Deidentified participant data
How to access data: Data can be requested by emailing the corresponding author at a.el.sohemy@utoronto.ca
When available: With publication

Supporting Documents
Document types: None

Additional Information
Who can access the data: Researchers whose proposed use of the data has been approved.
Types of analyses: For specified purposes
Mechanisms of data availability: After approval of proposal with a signed data access agreement and with investigator support.
